# Inter-brain synchrony during mother–infant interactive parenting in 3–4-month-old infants with and without an elevated likelihood of autism spectrum disorder

**DOI:** 10.1093/cercor/bhad395

**Published:** 2023-10-26

**Authors:** Yasuyo Minagawa, Masahiro Hata, Eriko Yamamoto, Daisuke Tsuzuki, Satoshi Morimoto

**Affiliations:** Department of Psychology, Faculty of Letters, Keio University, 4-1-1 Hiyoshi, Kohoku-ku, Yokohama 223-8521, Japan; Human Biology-Microbiome-Quantum Research Center (WPI-Bio2Q), Keio University, 35 Shinanomachi, Shinjuku-ku, Tokyo 160-8582, Japan; Center for Advanced Research of Logic and Sensibility, Global Research Institute, Keio University, 2-15-45 Mita, Minato-ku, Tokyo 108-8345, Japan; Center for Advanced Research of Logic and Sensibility, Global Research Institute, Keio University, 2-15-45 Mita, Minato-ku, Tokyo 108-8345, Japan; Center for Advanced Research of Logic and Sensibility, Global Research Institute, Keio University, 2-15-45 Mita, Minato-ku, Tokyo 108-8345, Japan; Department of Information Science, Faculty of Science and Technology, Kochi University, 2-5-1 Akebono-cho, kochi-shi, Kochi 780-8072, Japan; Center for Advanced Research of Logic and Sensibility, Global Research Institute, Keio University, 2-15-45 Mita, Minato-ku, Tokyo 108-8345, Japan

**Keywords:** attachment, autism spectrum disorder, breastfeeding, functional near-infrared spectroscopy, orbitofrontal cortex

## Abstract

Maternal bonding for mammalian infants is critical for their survival. Additionally, it is important for human infants’ development into social creatures. However, despite the ample neurobiological evidence of attachment for the mother’s brain, the interplay of this system in infants is poorly understood. We aimed to identify the neural substrates of synchrony in mothers and infants under three interactive conditions and compare the differences between groups with (*n* = 16) and without (*n* = 71) an elevated likelihood of autism spectrum disorder by examining the inter-brain synchrony between mothers and their 3–4-month-old infants. Mother–infant hyperscanning with functional near-infrared spectroscopy was performed during breastfeeding and while each of the mother and experimenter was holding the infants. The results showed almost no group differences, with both groups demonstrating the strongest inter-brain coupling for breastfeeding. The cerebral foci underlying these couplings differed between mothers and infants: the ventral prefrontal cortex, focusing on the right orbitofrontal cortex, in the mother and the left temporoparietal junction in the infant were chiefly involved in connecting the two brains. Furthermore, these synchronizations revealed many significant correlations with behavioral measures, including subsequent language development. The maternal reward-motivational system and the infant’s elementary mentalization system seem to underlie mother–infant coupling during breastfeeding.

## Introduction

Mammalian infants require early attachment to their caregivers for secure development because of their extreme prematurity at birth. For human infants, parental attachment contributes substantially to the cognitive development, fostering essential learning processes. One major example of such a process is social learning. Infants learn emotional processing of themselves and others by receiving and producing facial and vocal–emotional expressions through affiliative and affective interactions between caregivers and infants. These learning processes are sometimes unidirectional but mostly bidirectional. Infants acquire their first social skills to cope with outgroup members in the social world through mutual exchanges of social cues (such as gaze and touch) with their parents. Thus, social brain networks are constructed first in relationships with parents. Alternatively, the social-communicative brain is first shaped by parents’ interactions, enabling a child to bond with larger social groups such as friends, strangers, and individuals from other cultures (Bowlby 1978; Feldman 2015).

One of the significant pieces of evidence on the parent–infant relationship as a primitive foundation of social learning is the biobehavioral synchrony observed between parents and infants. According to Feldman (2017), biobehavioral synchrony consists of four systems: behavioral, autonomic, hormonal, and brain. Numerous researchers for half a century have investigated behavioral synchronization between parents and children. These behavioral entrainments include facial expressions, eye movements, vocalization, and touch (Jaffe et al. 2001; Feldman 2006; Cirelli 2018; Niedzwiecka et al. 2018). In most studies on experimenter-infant and parent–infant dyads, concurrent or sequential entrainment of these signals is observed, and larger entrainment has better outcomes for subsequent cognitive development. For example, concurrent synchrony of movement promotes prosocial behavior (Cirelli 2018). Subsequent studies further revealed an entrainment of autonomic (Van Leeuwen et al. 2003, 2009; Suga et al. 2019) and hormonal systems (such as oxytocin and cortisol secretion) that coordinate behavioral entrainment (Feldman et al. 2011a; Feldman 2012). Among the endocrine substances that regulate human behavior, oxytocin is well-established as a fundamental hormone crucial for parent–infant attachment and parenting. Various types of perceptual stimuli of infants via olfactory, visual, and auditory modalities were reported to induce maternal oxytocin elevation (Minami et al. 2022; White-Traut et al. 2022), which facilitates parenting. Regarding synchronized behavior, Feldman et al. (2011b), through the meticulous observation of parent–infant interactions using micro-behavior coding, demonstrated the involvement of oxytocin in both plasma and saliva in fostering interactive synchronized behavior and contingent parenting. Furthermore, this robust parent–infant synchrony was correlated with elevated oxytocin levels in infants (Feldman et al. 2010).

Regarding brain entrainment, brain synchrony, and the direct neural relationship between the mother and infant have not been sufficiently studied partly due to the technical problems in obtaining measurements for their brains simultaneously (hyperscanning) until recently. However, with the rapidly accumulating knowledge and evidence for adult brain coupling, hyperscanning of the developing population using electroencephalography (EEG) or functional near-infrared spectroscopy (fNIRS) has emerged as a viable approach. The literature on adult brain coupling has identified various types of interpersonal neural synchronizations concerning various social activities. Some of these studies discovered evidence of two brains working as one system (“two-in-one-system”) (Schilbach et al. 2013; Koike et al. 2015; Xu et al. 2023). Brain networks engaged in such neural coupling can be categorized into at least two streams: the mirror neuron system (MNS) (or action-observation network) (Hari and Kujala 2009) and the mentalization (MENT) network (Frith and Frith 2006). These two systems connect two (or more) brains to function as one system (Schilbach et al. 2010, 2013; Konvalinka and Roepstorff 2012; Minagawa et al. 2018). Such neural coupling in a “two-in-one system” enhances closeness, liking, empathy, cooperative behavior, and prosocial behavior (Babiloni et al. 2012; Toppi et al. 2016) among partners.

A mature social brain that can connect to other brains for better interaction can be hypothesized to develop according to the quality and quantity of parent–infant interactions at an early developmental stage (Feldman 2017). Studies on the developing population have primarily used EEG–EEG or fNIRS-fNIRS concurrent recording in children (Dikker et al. 2017; Reindl et al. 2018; Nguyen et al. 2020), in contrast to the relatively limited studies on infants. Reindl et al. (2018) demonstrated that the dorsolateral prefrontal cortex (dlPFC) and frontopolar cortex showed strong synchrony between parents and 5–9-year-old children during the joint condition of game playing using the higher spatial resolution of fNIRS. This synchronous activity was associated with emotional regulation in parents and children, according to questionnaire results. Other fNIRS studies have also reported that cooperative or combined conditions result in stronger neural coupling than that with the control condition between parents and children (Reindl et al. 2018; Miller et al. 2019; Nguyen et al. 2020).

In comparison with these studies in children, fNIRS hyperscanning data for infants are relatively scarce, as stated previously, although some studies (Minagawa et al. 2018; Piazza et al. 2020; Nguyen et al. 2021) have reported neural entrainment during early development. Piazza et al. (2020), for example, performed hyperscanning of 9–15-month-old infants interacting with an experimenter and identified the coupling of the medial part of the prefrontal areas during these interactions. Infant–adult concurrent measurements are also limited in the EEG modality; however, some of these studies showed alpha or theta band entrainment between the experimenter and infants (8–12 months old), which are related to social learning or attention systems (Leong et al. 2017; Wass et al. 2018). However, among these studies employing fNIRS or EEG, there is a scarcity of studies investigating parent–infant interactions (Minagawa et al. 2018; Nguyen et al. 2021). Therefore, this study centered its attention on infants, notably a substantial cohort of infants aged 3–4 months, to hyperscan with their parents.

The general findings, introduced above, of the previous hyperscanning studies on children can be summarized as follows: weaker parent–child entrainments are associated with poorer social attachment and cognitive consequences (Reindl et al. 2018; Nguyen et al. 2020; Azhari et al. 2021). Consistent with this finding, atypical social skills observed in children with autistic spectrum disorder (ASD) could be partly attributed to reduce developmental entrainment. A considerable amount of research on this issue has been conducted, and the findings have confirmed that children or adults with ASD tend to show weaker biobehavioral interpersonal synchrony than do typically developing children or adults (Tanabe et al. 2012; McNaughton and Redcay 2020). Reiterating, as previously mentioned, elevated oxytocin levels in both infants and parents have been linked to increased synchronized behavior (Feldman et al. 2010). This implies that children with ASD or infants with an elevated likelihood of ASD (EL infants), who are presumed to have atypical oxytocin levels and functioning (Moerkerke et al. 2021), potentially influenced by varying genetic factors, might exhibit reduced synchronization. However, this does not mean that they do not show any biobehavioral synchrony because previous studies have consistently identified synchrony in individuals with ASD in some conditions. In fact, individuals with ASD show high coherence comparable to that of the typically developing group for some motor and conversational synchrony tasks (Branigan et al. 2016; Koehne et al. 2016). With respect to parent–child synchrony, reduced synchrony has been reported for behavioral, autonomic, hormonal, and brain systems (Fitzpatrick et al. 2017; Saxbe et al. 2017). For example, children with ASD showing less electrodermal synchrony with their parents have higher degrees of ASD symptoms (Baker et al. 2015). A parent–child hyperscanning study performed using magnetoencephalography in children with ASD showed a positive correlation of mu suppression during an interaction (hypothesized index of mirror system activity) between the parent and child (Hasegawa et al. 2016). Furthermore, the degree of mu suppression was negatively correlated with autistic symptoms in mothers and children. In contrast, fNIRS hyperscanning did not show such ASD-specific neural tendency during a joint game with a parent (Kruppa et al. 2021). To our knowledge, no study has examined biobehavioral synchrony with parents in infants with an elevated likelihood of ASD (EL infants).

Our review suggested that the evidence for interpersonal neural entrainment through social interaction in the first year of life in typically developing (typical likelihood for ASD: TL) infants remains insufficient, with the evidence for EL infants being even more limited. Despite the significance of the parent–infant relationship in early development, studies performing concurrent measurements with caregivers are limited. Additional fNIRS studies on infants should be conducted to identify the precise brain regions and networks underlying parental interaction, such as the MENT and MNS. Moreover, using the hyperscanning method for younger infants would also yield valuable findings. To date, brain hyperscanning studies involving infants have primarily focused on those >8 months, partly because infants of this age have already started acquiring social skills. However, assessments of younger infants can be expected to reveal the developmental process of interactive social skills in addition to the primary form of inter-brain relationships, which may be shaped by parent–infant social interplay.

Consequently, this study aimed to reveal the neural basis of mother–infant synchrony in early development by examining a significant number of infants with typical likelihood of ASD (TL infant) during the most elementary forms of parenting activities: breastfeeding and holding infants. We aimed to identify the brain regions and networks involved in these parenting activities in both mothers and infants using a control separation condition. Additionally, we sought to gain a deeper understanding of how these mother–infant inter-brain couplings are associated with social cognitive behaviors and their attachment to explore the functions of inter-brain synchrony. Our secondary aim was to identify the differences in synchronized cortical activity between infants with EL and TL early in development. Hence, fNIRS hyperscanning was employed to examine hemodynamic activity and its couplings for 3–4-month-old infants (TL and EL) and their mothers under three conditions: breastfeeding, mother holding infant, and experimenter holding infant as a control condition. The two target conditions varied in the intensity of active interactive engagement. While we anticipate that the breastfeeding condition, characterized by highly interactive actions, will significantly enhance inter-brain synchrony, we posit that the holding condition, featuring fewer active actions, might still induce synchronization to some extent, drawing insights from the hyperscanning literature involving adults. In particular, previous studies have demonstrated that the brains of familiar dyads can synchronize during online interactions, even with tasks as subtle as maintaining eye contact (Tanabe et al. 2012; Koike et al. 2016). While no neuroimaging attempts have been made for hyperscanning infants with EL, these findings may reveal the neurocognitive origins of social interaction deficits. We hypothesize that the EL group may exhibit weaker brain synchronization, given the observed tendency of individuals with ASD to display attenuated biobehavioral synchronization, as reviewed above. This attenuation may be partly attributed to atypical genetic factors that could have already impacted early social development. Hyperscanning research has indicated that individual social brain networks connect with those of their counterparts to establish inter-brain synchronization. Consequently, an atypical social brain profile in the EL group (Dean 3rd et al. 2020) may encounter challenges in forming these cross-brain connections. From a technical standpoint, this study offered some notable advantages in its precise analysis of the coherence within these specific brain regions. Even though most hyperscanning studies with children or infants have examined coherence exclusively for identical brain regions within a dyad, this study examined the coherence of different brain regions by probabilistically identifying the brain regions using the virtual registration method. Finally, to reveal the relationship between neural synchronization and social cognitive traits, we performed longitudinal questionnaire assessments on attachment, depression, and social cognition and obtained behavioral video-recording data of the still-face paradigm (SFP) to correlate with brain data.

## Materials and methods

### Participants

Ninety-nine mother and infant dyads (TL infant group = 77, EL infant group = 22) participated in the experiment; however, 12 dyads were excluded because of fussiness or rejection of probe attachment and definition of EL infants. Regarding the definition of EL infants, initially, we included infants born preterm (< 35 weeks) but subsequently excluded those infants to refine the specificity of the EL group. Accordingly, the final dataset consisted of 71 dyads of mothers and infants (35 male infants) in the TL group and 16 dyads (10 male infants) in the EL group. The average age of the infants was 122.4 days (standard deviation [SD], 16 days) for TL infants and 122.4 days (SD, 16 days) for EL infants. The mothers were all native Japanese speakers, and none of the infants had neurological problems, according to the parental report. The average age of the mothers was 33.6 years (SD, 4.4 years) in the TL group and 35.8 years (SD, 4.1 years) in the EL group, with no significant difference (*t* = 1.66, *P* > 0.05). The EL group included infants with at least one older sibling with a clinical diagnosis of ASD. Out of the 71 TL infants, 40 did not have any siblings, resulting in a significant difference in the presence of siblings between the groups (*P* < 0.01, *χ*^2^ = 16.68). All infants, including EL infants, were within the average range of development, as measured by the Enjoji Scale of Infant Analytical Development (ESIAD) (Enjoji 1981). The two groups showed no statistically significant differences in the developmental quotient. TL infants had no family (first-degree relative) history of ASD. The study procedures were approved by the ethics committee of Keio University, Faculty of Letters (no. 150320103), and written informed consent was obtained from all parents.

### Experimental design

Hyperscanning sessions were conducted under three conditions: (i) breastfeeding condition, (ii) holding condition, and (iii) separation (control) condition. These sessions were performed in this order for three-fourths of the dyads for each group; for the remaining dyads, session (ii) or (iii) was conducted first, depending on the state of the infants. The sessions (ii) and (iii) involved mother–infant hyperscanning while the infant was being held; the infant’s mother held the infant in the holding condition while an experimenter (first author) held the infant during the control condition. Throughout these sessions, cortical hemodynamic activity in mothers and infants was measured in the same brain area to compare the amplitude of cortical synchronicity among the conditions and between the TL and EL groups. To evaluate the cognitive function of hemodynamic synchronization, the results of questionnaires on development, language acquisition, mother–infant attachment and temperament, and behavioral coding of mother–infant interactions assessed using the SFP were correlated with the synchronization data.

### fNIRS recordings

The bilateral temporal and frontal areas were measured using 44 channels for mothers and 3–4-month-old infants. NIRS (ETG-7000; Hitachi Medical Co., Japan) was used to measure hemoglobin (Hb) concentration changes in the optical paths in the brain between the emitter and detection probes separated by 2 cm (infant) and 3 cm (mother) on the scalp surface. This separation, which was adjusted for infants or adults, enabled us to measure hemodynamic changes in the brain 2.5–3 cm deep from the head surface (Fukui et al. 2003). This instrument emits two wavelengths (approximately 780 nm and 830 nm) of continuous near-infrared lasers, which are modulated at different frequencies depending on the channels and wavelengths and detected with sharp frequency filters of lock-in amplifiers (Watanabe et al. 1998).

### fNIRS localization

**Fig. 1:**
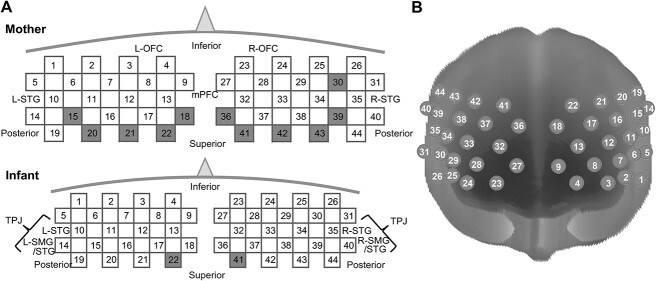
Channel locations in both the infant and mother, are presented in (A) top view and (B) 3D view. Channels not included in the final data set are indicated in gray.

The measurement areas and probe pads used were comparable between mothers and infants. To measure bilateral temporal, parietal, and frontal areas with 44 channels, two probe pads, each of which had eight incident and seven detection probes arranged in a transformed 3 × 5 grid (22 channels), were fitted on each side of the head using the international 10–20 system for both mother and infant. Specifically, two pads were jointly attached such that the joined vertical line was placed on the brain midline on the forehead, and the lowest lines of the probes were aligned to the T3-Fp1-Fp2-T4 line (Fig. 1). However, the separation distance between the emitter and detector was 30 and 20 mm for adults and infants, respectively, considering the difference in head size. Brain regions corresponding to the NIRS channels were estimated using virtual registration (Tsuzuki et al. 2007). Since this placement was not a standard approach in which we connected two probe pads joined in the midline, we estimated the brain region by analyzing the head size data (infants, 413 mm) of the participants by applying the virtual registration method to precisely estimate the brain regions for each channel. For this estimation, the length between the adjacent probes across the two probe pads (right and left pads) was 37 and 25 mm for adults and infants, respectively (Fig. 1).

### Questionnaires and behavioral testing of attachment

ESIAD, the most popular developmental questionnaire in Japan (Enjoji 1981), was employed to confirm general development or correlate language scores (LS) developments with the fNIRS data. In addition, the Japanese versions of the Postpartum Bonding Questionnaire (PBQ) (Brockington et al. 2006), Temperament and Character Inventory (TCI) (short version), and Preschool TCI (PSTCI) (temperament questions only) (Sugawara et al. 1999; Constantino et al. 2002) were used. Among the TCI questionnaires, we only focused on measures of reward dependency (RDT) for mothers (RDTm) and infants (RDTi) and cooperativeness for mothers. Language development was tested using the Japanese version of the MacArthur–Bates Communicative Development Inventories at 18 months of age (Ogura and Watamaki 2004). Across these measures, which encompassed both questionnaire-based assessments and behavioral tests, no significant differences between the two groups (see Supplementary Figs. S1 and S2).

The SFP is a structured experimental method to evoke infants’ reactions to responsive and unresponsive parents and assess their attachment. Interactions between mothers and infants were video recorded according to the SFP protocol (Tronick et al. 1978). We set the duration of each SFP episode as follows: 3 min of free-play, 2 min of still-face phase during which the mother ceased to respond, and 1 min of reunion (free-play). Two video cameras each were used to focus on the mother and infant.

### Procedures

fNIRS hyperscanning was performed under three conditions in a dimly lit, sound-attenuated room. The mother breastfed the infant in the first session after fNIRS pads were attached to her and the infant’s heads. After this session, the mother held the infant in her arms in a relaxed fashion without performing any task during the hyperscanning of the mother and infant’s brains. At this stage, infants were mostly drowsy, and mothers did not attempt specific interactions such as speaking to or smiling at the infants. They sometimes soothed the infants by gently moving them. Finally, one female experimenter (first author) held the infant to measure brain activity while the mother and infant were separated. Specifically, the mother and experimenter were separated by approximately 70 cm, and the mother was seated such that she did not see her infant held by the experimenter in the same room. Again, most infants were drowsy, and the experimenter soothed infants as the mothers did. Each session lasted at least 5 min and for a maximum of 10 min, depending on the presence of motion artifacts detected through visual inspections during data acquisition. On average, the session durations for TL and EL infants were as follows: 306 ± 87 and 336 ± 86 s for the breastfeeding condition, 310 ± 109 and 377 ± 118 s for the holding condition, and 267 ± 76 and 284 ± 26 s for the control condition.

Before or after the fNIRS session, depending on the infant’s condition, we conducted a session for the SFP and questionnaire assessments. We instructed mothers to interact with their infants according to the general SFP criteria (Tronick et al. 1978). We also asked the mothers to complete the questionnaires, as stated above.

### Data analysis

Since hemodynamic data obtained during interactions include systemic and motion artifacts, careful preprocessing procedures were performed by employing three steps for light-intensity data and three for hemoglobin data after conversion. First, usable sample periods of light intensity data were determined using the PHOEBE signal-to-noise ratio criterion (Pollonini et al. 2016). For the adult (mother) data, default settings were used for heart rate detection (i.e. a first-order Butterworth bandpass filter with a lower cutoff frequency of 0.8 Hz and an upper cutoff frequency of 1.95 Hz). On the other hand, for the infant data, we adopted lower and upper cutoff frequencies of 1.2 and 3.0 Hz, respectively. Following the PHOEBE application, the median ratio of valid data for mothers and infants was 0.69 (Interquartile Range [IQR] = 0.16) for the breastfeeding condition, 0.79 (IQR = 0.12) for the holding condition, and 0.77 (IQR = 0.14) for the control condition (see Supplementary Fig. S3). Wavelet filtering, which was implemented in Homer 2 as one of the preprocessing functions (hmrMotionCorrectWavelet), was then applied to exclude motion-related artifacts. Light intensity data with > 1.5 IQR for adults and 0.5 IQR for infants were regarded as artifacts (Behrendt et al. 2018). Finally, bandpass filtering (0.05–0.8 Hz) was applied using a third-order Butterworth filter.

After converting light intensity data to oxygenated hemoglobin (oxy-Hb) and deoxygenated hemoglobin (deoxy-Hb) data using the modified Beer–Lambert’s law, the Hb data were preprocessed with the hemodynamic modality separation method (Yamada et al. 2012) to eliminate systemic artifact data exclusively for adult data. Wavelet-MDL was applied to remove low-frequency noise (Jang et al. 2009). The Hb data were filtered with an AR-model-based prewhitening filter (Barker et al. 2013) to avoid biased detection sensitivity depending on the frequency band (Morimoto and Minagawa 2022) because the hemodynamic time course of brain responses is intrinsically different between adults and young infants owing to the immature vascular system in infants. The order of the AR filter was set to 100 (10 s).

The wavelet transforms coherence (WTC) (Grinsted et al. 2004) was calculated to evaluate the cross-correlation between the two-time series of fNIRS signals generated by the dyads as a function of frequency and time. The WTC is an index for synchronized brain activity between two persons (Cui et al. 2012). Each participant had 44 channels, and all combinations across and within the brain were analyzed. However, some channels had highly noisy data in many participants, mostly owing to insufficient light penetration; 11 channels for the mother’s data and 2 channels for the infant’s data were excluded from the final data set (see Fig. 1). This exclusion primarily resulted from the criterion that the median of the usable data period ratio assessed by PHOEBE (see Supplementary Fig. S3) was < 0.5. This resulted in 33 and 42 channels for mothers and infants, respectively. Thus, the WTC was calculated for 1386 channel pairs in each dyad for the between-brain analysis, while the within-brain analysis entailed 528 and 861 combinations for the mother and infant brains, respectively. The WTCs were averaged along the time domain, according to the method proposed by Zhang et al. (2020). In this study, we exclusively focused on the 0.05–0.09-Hz frequency band because (i) periodical systemic vascular signals are chiefly contained in a frequency of approximately 0.1 Hz, corresponding to the Mayer wave, (ii) the minimum duration of our data was approximately 5 min, which is insufficient for extracting slow-frequency data under 0.05 Hz, and (iii) our simulation test revealed good detection around this frequency band (Morimoto and Minagawa 2022). The averaged WTC value within 0.05–0.09 Hz for each channel pair in between-brain or within-brain connectivity was used for the analysis of variance (ANOVA) with the participant group and conditions as factors. To apply ANOVA, we excluded channel pairs with a data length shorter than 57.6 s, a criterion derived from the minimum data length required to compute 50 consecutive sample lengths of WTC at 0.05 Hz. As a result, certain channel pairs were omitted depending on the dyads and conditions, resulting in an average of 598.5 (SD:370) pairs per dyad for the breastfeeding condition, 1073.3 (SD:295) pairs for the holding condition, and 1159.0 (SD:277) pairs for the control condition. As we applied ANOVA to WTC of all channel combinations (1386), we employed false discovery rate (FDR) using Storey’s procedure (Storey 2002) to correct multiple comparisons. As the number of usable data differs depending on the channel combination; FDR was applied accordingly. In addition to conducting ANOVA to explore conditional differences, we also compared the Wavelet Transform Coherence (WTC) between actual dyad pairs and a baseline of WTC. Specifically, we generated phase-randomized surrogate data with identical lengths and identical missing value periods for each channel and each participant. Subsequently, we computed the mean WTC values within the frequency range of interest (i.e. 0.05–0.09 Hz) as we did for the real dataset. This calculation was repeated 1000 times to establish baseline null distributions. To gauge the relative WTC values of the actual dyads in relation to the baseline distributions, we computed z-scores for each channel combination and dyad. The significance of these z-scores for each channel combination was evaluated using one-sample *t*-tests. To address multiple comparisons, *P*-values were adjusted using the Holm–Bonferroni method.

Finally, correlations between mother–infant WTCs, during breastfeeding and the behavioral indices including the data obtained from the questionnaires and video-coding of SFP, were performed using Spearman’s correlation coefficients (uncorrected). These analyses were exclusively conducted for channel combinations that exhibited a significant main effect of the condition, all of which were attributed to the heightened strength of the breastfeeding condition compared to the other conditions. As this main effect was related to breastfeeding vs. holding and/or breastfeeding vs. control, the WTC values employed here represented the differences in WTC (subtracted value) between breastfeeding and one of the two conditions. It should be noted that the number of samples varied depending on the type of correlation analysis due to the absence of WTC data for particular channel combinations and also the number of behavioral or questionnaire data obtained in the follow-up study (see Supplementary Table S2).

### Video coding

Three coders analyzed the SFP videos using ELAN (Brugman and Russel 2004). For each phase of free-play and still-face, we coded the mother’s smile and touch and the infant’s vocalization, and crying, including fussy vocalization. Assessment of inter-coder agreement for 25% of the data coded by each of the three coders showed an inter-rater agreement of 0.79 for the mother’s data and 0.77 for the infant’s data.

## Results

**Fig. 2:**
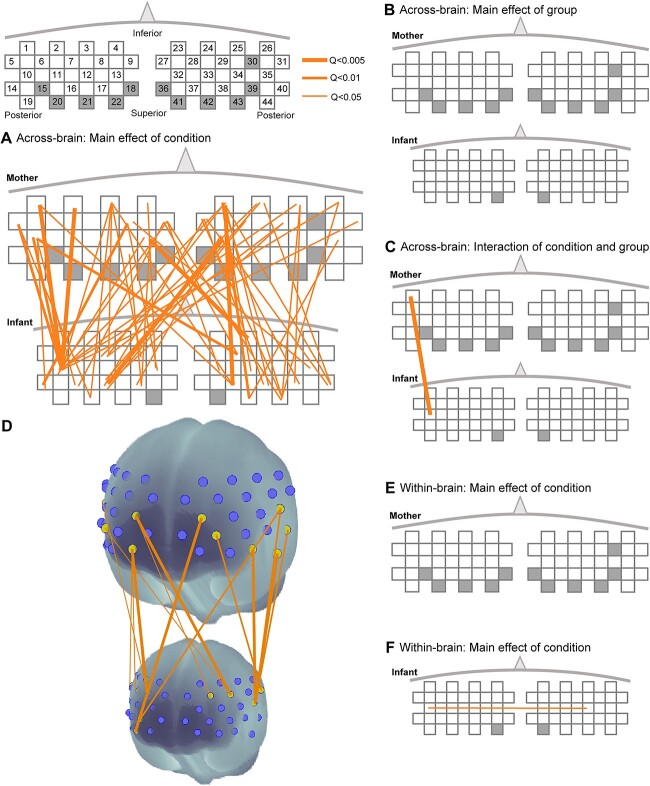
Significant brain synchrony between the mother and infant. Results of ANOVA for across-brain WTCs (synchrony) are indicated for the main effects of (A) condition and (B) group, and (C) their interaction. (D) The top 15 strongest WTCs among the 57 significant connections are indicated. Results of ANOVA for within-brain WTCs (functional connectivity) are shown for (E) the mother’s brain and (F) the infant’s brain.

To examine inter-brain synchrony among the three experimental conditions (i.e. breastfeeding, mother holding infant, and control) in relation to the group (i.e. TL and EL infants), the average WTC was analyzed using ANOVA for each channel pair (Ch-pair). The results for between brain synchrony (Fig. 2A–C) revealed a significant main effect of the condition on many Ch-pairs owing to the stronger synchrony during the breastfeeding condition. After rigid correction for multiple comparisons, 57 connections were significant, all of which were stronger for the breastfeeding condition than for the other condition(s) (FDR: Q < 0.05) (Fig. 2A, see Supplementary Table S1 for statistics). Figure 2 D shows the top 20 strongest synchronizations among the 57 significant ones. The strongest connection was between maternal (M) Ch13 (left [L]-frontal pole [FP]) and infant (I) Ch24 (right [R]-orbitofrontal cortex [OFC]) (F(2, 137) = 14.06, *P* = 0.000002, Q = 0.0018, partial *η*^2^ = 0.17) (Fig. 2A and D). The mothers’ left middle temporal gyrus (MTG) and left temporal pole (TP) (M: Ch1, 2) also synchronized strongly with the infants’ left superior temporal gyrus (STG) (I: Ch10) (Q = 0.04). In contrast to our expectation, no Ch-pairs showed a significant main effect on the groups, indicating that brain coupling for the EL dyads was not weaker than that for the TL group. Rather, the EL group showed significantly stronger synchronization for M: Ch1—I: Ch10 (MTG—STG) than the TL group for the breastfeeding condition, as indicated by the significant interaction between group and condition (F(2, 133) = 9.38, *P* = 0.00015, Q = 0.0123, partial *η*^2^ = 0.12). Similar ANOVAs performed for within-brain synchronization (functional connectivity) for each mother and infant (Fig. 2E and F) revealed a significant main effect (F(2, 154) = 10.64, *P* = 0.00004, Q = 0.041, partial *η*^2^ = 0.12) for only one connectivity (Ch10–Ch34) in the infant brain, which was because of the stronger connectivity for the breastfeeding condition. Some factors, including the presence of the infant’s siblings and the order of the session condition, were not completely controlled in the present experiment. Thus, WTC for the breastfeeding condition was compared for each factor including gender difference that was balanced. No statistically significant differences were found for any channel pairs associated with these factors (Supplementary Fig. S4).

In addition to the conditional comparisons, we conducted an analysis of WTCs relative to a baseline WTC (see Supplementary Fig. S5). In all three conditions, we observed significantly stronger WTCs, with the breastfeeding condition exhibiting more significant channel pairs than any of the other conditions, aligning with the patterns observed in the ANOVA results.

**Fig. 3:**
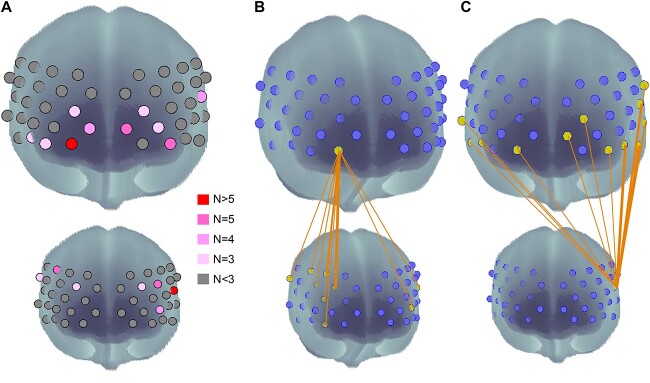
Brain regions showing more significant WTCs than the counterpart’s brain. (A) Number of significant connections from one channel for each mother and infant. (B) Nine significant WTCs are connected to the mother’s R-OFC channel. (C) Twelve significant WTCs are connected to the infant’s L-TPJ.

Figure 3 clarifies the brain regions that showed greater involvement among the 57 significant brain couplings during breastfeeding. The number of significant synchronizations from each Ch is shown in different colors for the mother (Fig. 3A top) and infants (Fig. 3A bottom). Each brain has different strong seeds showing many connections with the counterpart’s brain. For instance, the mothers showed more synchronizations in the ventral part of the midline area with a primary focus on the R-OFC (Ch24, Fig. 3B) and a secondary focus on the medial prefrontal cortex (mPFC) (Ch9, 27). In contrast, infant synchronization was more frequently observed in the temporoparietal areas. Specifically, Ch10 (L-STG), which can also be categorized into the temporoparietal junction (TPJ), showed 12 significant connections (Fig. 3C), in addition to Ch40 (R-TPJ). Channels in the dlPFC (I: Ch16, 17, 43) are also involved in this process.

**Fig. 4:**
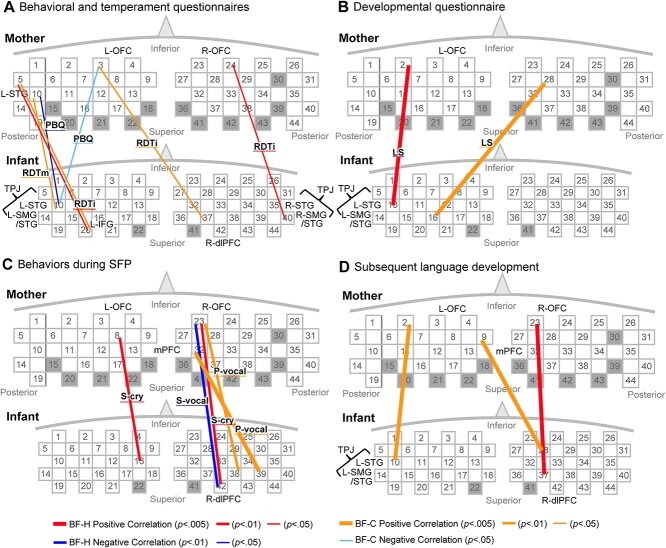
Correlations between brain synchrony and various behavioral measures (A-D). These measures are (A) questionnaire-based measures, including reward dependency in mothers (RDTm) and infants (RDTi), and the parental bonding quotient (PBQ); (B) language score (LS) at 3–4 months of age; (C) behavioral coding results during SFP, including vocalization during the free-play phase (P-vocal), vocalization during the still-face phase (S-vocal), and crying or fussing during the still-face phase (S-cry); (D) score for expressive vocabulary at 18 months of age.

Figure 4 shows the analysis results for the association of these inter-brain synchronizations during breastfeeding against the holding or control condition, with the mother–infant relationship such as the infant’s temperament, and social behavior (see Supplementary Table S2 for detailed statistics). In the figures, the correlation results for breastfeeding vs. holding and breastfeeding vs. control were depicted in different colors; however, the robust WTCs observed during breastfeeding, as indicated by the significant main effect, significantly differed from both the holding and control conditions across most channel pairs (see Supplementary Table S1). Moreover, there were no significant differences in WTCs between the holding and control conditions. Consequently, we interpret the holding condition as serving as an additional control condition when evaluating breastfeeding-related WTCs, and we do not make a distinct differentiation between the WTC results from these two comparisons. In fact, these two scenarios largely yield similar outcomes (e.g. Fig. 4A and I: Ch20-M: Ch5, Fig. 4B, D, I: Ch2-M: Ch10). Figure 4 ( A and B) indicates significant correlations (*P* < 0.05, uncorrected) between mother-infant synchrony and questionnaire results. Strong correlations were observed in four connections from the infants’ L-STG (I: Ch10-M: Ch2, 3, 10). Among them, three had emotion-related correlations (Fig. 4A), such as those with PBQ, reflecting mother–infant attachment and RDTm, which is an index of the mother’s emotional sensitivity. Specifically, two synchronizations between the infant’s L-STG (Ch 10) and the mother’s L-STG (Ch10) or L-OFC (Ch 3) showed a negative correlation, indicating more difficult attachment associated with weaker synchronization. Mother’s sensitivity (RDTm) showed a positive correlation with WTC between L-STGs for the mother and infant. The remaining correlation pertains to the language score (LS) (Fig. 4B), and no significant correlations were observed for the scores related to motor and social developments. Apart from the infant’s L-STG-related synchronizations, three significant correlations were associated with RDTi, which reflects the infant’s emotional sensitivity. For example, infant R-supramarginal gyrus (SMG)/STG synchronizing to mother R-OFC/mPFC showed a significant correlation with RDTi. Contradictory to our expectation, there were no correlations between WTCs and the mother’s contingent responsiveness during free play (SFP). Assessment of correlations was performed between mother–infant synchronization and behavioral measures during the SFP (Fig. 4C), revealing that the R-OFC of the mother had many correlations. Crying (fussiness) of infants during the still phase reflected mother–infant attachment; two significant correlations were observed between cry duration and synchrony (Fig. 4C). Labelling of “vocalization” mostly reflected positive vocalization because it did not include “cry” or fussy vocalization. Such vocalization during the still phase negatively correlated with the connection between the mother’s R-OFC and the infant’s R-dlPFC. In contrast, positive vocalization during the “free-play” phase (P-vocal) positively correlated with a similar R-OFC-related synchronization (I: Ch38-M: Ch23). Finally, the correlations between mother–infant synchronization and subsequent language development (expressive vocabulary) of infants at 18 months showed three significant correlations (Fig. 4D). Among them, two were related to the mother’s R-OFC and the infant’s L-STG. A significant correlation obtained for synchronization between the infant’s L-STG and the mother’s temporal pole (I: Ch10-M: Ch24) was comparable with a correlation observed for the LS score at 6 months old (Fig. 4B). This suggests a consistent influence on language development.

## Discussion

This study examined inter-brain synchrony early in development during breastfeeding and holding, which are very basic forms of mother–infant interaction. The primary goals were to reveal the neural basis of mother-infant synchronization by identifying the precise cortical regions and functions in the brains of mothers and infants and identify differences between TL and EL infants at 3–4 months of age. We observed strong inter-brain synchrony exclusively during breastfeeding; however, no differences were observed between the two infant groups. Dyads of TL and EL groups showed synchronization foci around the ventral part of the frontal midline for the mothers and bilateral TPJ areas and other frontal areas, that is, the inferior frontal gyrus (IFG) and dlPFC. Some of these inter-brain synchronies were significantly correlated with indices of attachment, and social cognitive tendencies. Furthermore, some of these connections predicted subsequent language abilities. These findings demonstrate a fundamental neural system of mother–infant synchronization related to the basic but critical caretaking behavior of breastfeeding. Moreover, the TL and EL infants shared these neural underpinnings of synchronization during this period. Hence, we present the inaugural evidence of inter-brain synchrony in young infants, including those with an elevated likelihood of ASD.

### Group differences

While one of the main objectives of this study was to examine whether the early neural substrates of interpersonal synchrony differed between EL and TL infants, the results indicated no group differences. EL infants showed larger synchronization for the L-MTGm–L-STGi connection, a typical connection between mothers and infants. EL infants, who were considered to have an atypical genetic background, were hypothesized to have weaker synchronization considering the previous literature; however, they did not exhibit any significant differences in breastfeeding-related neural couplings as long as 3–4 months from birth. The inter-brain synchrony in the infant involved the TPJs (STG, STS, and SMG) on both sides, particularly the left side. The TPJ is a key region involved in attributing mental states and intentions to others (Saxe and Kanwisher 2003; Kubit and Jack 2013). The posterior STS/STG within the TPJ processes the movement and action prediction of others (Grossman and Blake 2001; Michels et al. 2009). The R-TPJ is the dominant region for such social processing; however, the L-TPJ has been reported to play a similar role (Perner et al. 2006; Schurz et al. 2014). Thus, 3–4-month-olds are assumed to utilize the left and right TPJ as a very primitive form of mentalization. At this point, this processing may not be particularly specialized in human social beliefs or intentions; however, infants can adjust their sucking behavior to the mother’s feeding behavior by anticipating the mother’s movement through basic breastfeeding routines. We will discuss this point later, but it is likely that at this stage, the functional localization of the TPJ has not been completed, and breastfeeding does not necessarily require socially relevant predictive coding skills that are usually processed at the R-TPJ, resulting in similar neural processing in the two groups. This interpretation may be partly supported by the findings of previous studies on adults and children with ASD. While many studies have reported reduced synchronization in individuals with ASD, the level of synchronization in specific tasks, such as motor synchrony (e.g. torso movement and tapping) and conversational synchrony (e.g. alignment of syntax with an experimenter’s syntax), is not substantially different between individuals with ASD and neurotypical individuals (Allen et al. 2011; Nadig et al. 2015; Koehne et al. 2016; Noel et al. 2018). Similarly, a recent systematic review examining synchronization across different sensory modalities (Murat Baldwin et al. 2022) found no conclusive differences in visual-motor synchronization, which is relevant to this study. However, it did reveal weaker synchronization in ASD when it comes to cross-perceptual modality tasks, such as audio-visual or visual-tactile integration. According to these reports (McNaughton and Redcay 2020; Murat Baldwin et al. 2022), individuals with ASD, both children and adults, can still adjust their movements to those of others using visual or tactile stimuli for certain motor tasks. However, this does not imply that individuals with ASD are entirely comparable to the neurotypical population in terms of motor synchrony. We see evidence of weaker motion synchrony performance in individuals with ASD, particularly in more complex and socially related tasks (McNaughton and Redcay 2020; Murat Baldwin et al. 2022).

Among the limited studies on neuronal measures for ASD, a recent fNIRS study (Kruppa et al. 2021) showed no differences in hemodynamic synchronization between children with ASD and typically developing children during a joint button-press task. This result supports our interpretation because their task, which required simple motor adjustment but not advanced social skills, elicited similar neural synchronization in the two groups. EL infants could employ a similar neuronal basis as TL infants for breastfeeding, which is a basic but crucial interplay with their mother for survival because breastfeeding in this experiment is unlikely to require higher mentalization skills between the dyad.

Another point worth discussing about EL infants is the stronger synchronization (significant condition × group interaction) between the mother’s L-MTG and the infant’s L-STG during breastfeeding. We hypothesize that this phenomenon might be attributed to the heightened sensitivity of mothers with EL infants. Because EL mothers already had a child with ASD, they would have experienced some ASD-related difficulties and worries about their children, resulting in higher maternal sensitivity to their younger children. Indeed, significant synchronization between similar brain areas was positively correlated with mothers’ emotional sensitivity (RDTm), suggesting that the mother’s side strongly influenced this synchronization. Previous mother–child studies showing higher physiological synchrony with their children in anxious mothers (Smith et al. 2022) further support our interpretation.

However, it is essential to acknowledge the possibility that the group similarity observed in this study may be influenced by biased sampling or the relatively small number of our EL infants included. Specifically, it is possible that our final cohort of EL infants happened to develop without exhibiting any autistic symptoms. To explore this possibility, we attempted to follow their development at the age of 3 years using the Kyoto Scale of Psychological Development (Ikuzawa et al. 2001), a standardized general developmental scale assessed by behaviors for Japanese children.

Nevertheless, some infants in our study happened to be absent at measurement for 3 years old, and others dropped out from our cohort due to the challenges posed by the COVID-19 pandemic. Among the nine infants we were able to follow, two exhibited notably delayed development, particularly in language and social aspects. It is worth noting that in Japan, ASD symptoms can be diagnosed after 3 years of age at the earliest, and as a result, these two infants have not yet received an official diagnosis. However, the developmental tendencies observed in our EL group may align with previous research findings, as siblings of individuals with ASD have a likelihood of 10–30% of developing ASD themselves.

Furthermore, we have valid reasons to believe that our present EL group is not biased in terms of risk factors. These EL infants exhibited discernible developmental differences from a TL group in various experiments, including an eye-tracking study focused on facial movement and another fNIRS study examining responses to their mother’s and a stranger’s speech (Minagawa et al. 2023). It’s important to note that these differences were observed when the infants were 6 months old, indicating that developmental distinctions may not be as pronounced at 3–4 months of age. For future studies, it’s crucial to carefully consider this sampling issue by either including more EL infants or analyzing EL infants who later develop ASD.

### Condition differences: Strong synchronization during breastfeeding

Among the three conditions (breastfeeding, holding, and control), breastfeeding exclusively showed significantly stronger inter-brain synchrony between the mother and infant. Adult–infant hyperscanning studies with EEG or fNIRS have revealed that social interaction induces stronger neural coupling within dyads (Piazza et al. 2020; Wass et al. 2020). These synchronizations are associated with social signals, such as eye gaze and touch (Leong et al. 2017; Piazza et al. 2020; Nguyen et al. 2021). Consequently, most of these studies examined older infants (older than 7 months) who had mature skills in processing social signals. This study, targeting younger infants during their maternal interactions, revealed that even young infants aged 3–4 months show strong brain synchronization with their mothers. Moreover, unlike most previous studies that only examined synchrony between the same brain areas (e.g. mother’s IFG and infant’s IFG), we analyzed the WTC between different brain areas. We further employed the virtual registration method to precisely estimate the brain regions engaging in synchronization in mother and infant brains. Indeed, these analyses identified the different neural foci of mother–infant synchrony during breastfeeding, R-OFC for mothers and L-TPJ for infants.

Here, we discuss the functions of these areas with strong synchronizations by considering the results of the correlation analysis with behaviors (Fig. 4). The mother’s brain areas exhibiting prominent synchronization were the ventral part of the prefrontal area, including the OFC, mPFC, and frontopolar area (Fig. 3A). The most prominent channel was the R-OFC (Fig. 3B), which is primarily the cerebral base of attachment as a part of the reward network. As initially shown in animal studies (Young and Wang 2004; Moriceau and Sullivan 2005; Goursaud and Bachevalier 2007), the OFC is critically involved in attachment behavior in human infants and mothers. Neuroimaging studies with fMRI and fNIRS consistently reported that the OFC, including the anterior part of the OFC, plays an important role in decoding attachment-related positive affect not only for mothers (Leibenluft et al. 2004; Nitschke et al. 2004; Minagawa-Kawai et al. 2009), but also for infants (Minagawa-Kawai et al. 2009). Correlation analysis with behavioral measures during the SFP revealed that inter-brain coupling from the mother’s R-OFC and infant’s dlPFC was significantly correlated with negative vocalization during the still-face phase and with positive vocalization during the free-play phase (Fig. 4). Because more negative vocalizations such as crying and fussy voice during the still-face reflect stronger attachment amplitude (Tronick et al. 1978), The R-OFC–dlPFC synchrony is thought to be critically related to mother–infant attachment. The positive correlation between the same synchrony and positive vocalization during the “free-play” phase also supports this interpretation. Moreover, another R-OFC-related synchrony connected to the infant’s R-TPJ exhibited a significant correlation with infants’ emotional sensitivity, as indexed by RDT. These correlations with SFP and RDTi indices strongly support the view that OFC-related mothers’ synchrony with infants’ brains during breastfeeding reflects the neural foundation of mother–infant attachment. This may also be elicited by the mothers’ emotional state of appraising affective value. Such attachment-related motivation is likely to facilitate the socially interactive behavior of mothers toward their infants which eventually leads to higher infant language and social skills. This was suggested by positive correlations between the three synchronizations and the expressive language score at 18 months of age and those between two synchronizations and the language and social scores at 6 months of age (Fig. 4B and C).

The R-OFC (Ch 23, 24), from which many synchronizations were observed in the mothers, corresponds to the anterior part of the OFC captured by fNIRS. As proposed by Feldman (2017), these OFC activations together with the mPFC are part of the “reward-motivation system,” which includes the striatum, amygdala, anterior cingulate cortex (mPFC), and OFC. This system is largely supported by the subcortical limbic system, which cannot be detected using fNIRS with limited optical emission. Therefore, during the breastfeeding condition, the striatum within this system, including the caudate, putamen, and amygdala, may have shared similar activities as the OFC. However, among these systems, the OFC, the endpoint of the reward path structure after a long history of human evolution (Stalnaker et al. 2015), plays a central role in human attachment. It is a hub, facilitating parenting by predicting and orienting infants’ signals, coordinating appropriate behaviors, and coding and appraising affective values (Kringelbach 2005; Parsons et al. 2013) to foster human social-communicative ability. Thus, such a critical area can be expected to connect to the infant’s brain during breastfeeding during early development. Most of the existing studies discussing reward-motivation systems primarily focused on the activity of the mothers’ OFC; however, the infants’ OFC also plays a significant role. The second strongest synchrony during breastfeeding was observed between the mother’s frontopolar and the infant’s R-OFC. Thus, the human reward-motivation system is functional in 3–4-month-old infants.

As previously mentioned, the most prominent brain area for breastfeeding-related neural coupling on the infant side was the L-TPJ. More precisely, Ch10, corresponding to L-STG/STS, which can be regarded as part of the TPJ, had the largest number of synchronizations with the mother’s fNIRS channels. This area (Ch10) also shows within-brain synchronization, namely functional connectivity with the right IFG channel during breastfeeding (Fig. 2). As already discussed, these TPJ synchronizations may entail predicting the mother’s movement to coordinate breastfeeding actions. Although the left side of the TPJ was dominant in the present study, these synchronous activities are considered to be supported by the early form of the mentalization system in infants. One piece of evidence supporting this interpretation is the multiple significant correlations between L-TPJ-related synchrony and behaviors, including two correlations for the bonding amplitude (PBQ) (Fig. 4B). The mother-infant bonding amplitude and the infant’s primitive mentalization ability could influence this synchrony, which originated from the infant’s L-TPJ because the PBQ partly reflects an infant’s social behavior, including the emotional aspect. Apart from the PBQ, the mother’s sensitivity, as indicated by RDTm, also showed a significant correlation with L-TPJ-related synchrony (L-TPJm–L-TPJi). This indicates that infants’ mentalization ability, the mother’s sensitivity, and the resultant mother-infant bond are associated with these L-TPJ-related synchronies. Such an early bond may shape an infant’s social-communicative brain, as shown by the significant correlations between L-TPJ-related synchrony and subsequent vocabulary scores and language and social scores at 3–4 months of age (Fig. 4B and C).

Oxytocin may have played a role in promoting higher mother–infant brain synchrony during the breastfeeding period. Triggered by the infant’s sucking behavior, oxytocin, a neuropeptide hormone, is produced in the hypothalamus and controls the milk ejection. Increment of peripheral endogenous oxytocin concentration during breastfeeding has been observed in both saliva and plasma (McNeilly et al. 1983; White-Traut et al. 2009). Because oxytocin levels have been reported to positively correlate with affective synchrony between parents and infants (Feldman et al. 2010; Feldman et al. 2011a), we can speculate that such elevation of oxytocin might have facilitated synchronization in this study. However, it’s important to note that infant stimuli alone, without active interaction, can also trigger an increase in oxytocin (Minami et al. 2022). Some studies have reported no significant differences in parental oxytocin levels between holding or baseline conditions and the breastfeeding condition, although the results in the literature are inconsistent (McNeilly et al. 1983; White-Traut et al. 2009; Matsunaga et al. 2020; Nagahashi-Araki et al. 2022). Therefore, we cannot conclusively assert that the elevation of oxytocin directly enhanced brain synchrony in this experiment. Nevertheless, it is evident that oxytocin plays a crucial role in the formation of biobehavioral synchrony during parent–infant interactions in daily life, and the interbrain synchrony demonstrated through fNIRS hyperscanning in this study may represent one aspect of this biobehavioral synchrony.

Before finalizing this section, we would like to highlight another targeted condition: the holding condition. We hypothesized that the holding condition would elicit stronger synchronization owing to the mother’s physical contact and olfactory factors than the control condition, in which the experimenter held the infant. Some previous studies have reported stronger synchrony during proximal conditions, such as holding (Minagawa et al. 2018; Nguyen et al. 2021), than distal conditions. One reason for this difference may be the difference in the frequency bands analyzed for the WTC. Specifically, this study employed strict criteria in choosing the frequency band of the WTC (i.e. 0.05–0.09 Hz) to exclude systemic effects. This suggests that holding conditions could induce synchronization of systemic physiological responses, such as heartbeat and respiration.

### Technical issues and limitations

fNIRS hyperscanning in adults and infants is an emerging field that has still not matured regarding technical issues. These issues include contamination of systemic signals under interactive activity and differences in intrinsic hemodynamics between adults and young infants with developing neurovascular systems. We have carefully addressed these issues by performing simulation studies and adult hyperscanning (Morimoto and Minagawa 2022; Xu et al. 2023. One of these findings indicated contamination by breathing-related factors and breathing synchrony affects hemodynamic WTC around 0.15–0.5 Hz. Consequently, we chose a frequency band of 0.05–0.09 Hz by avoiding the Mayer wave. Choosing the appropriate frequency is critical for hyperscanning studies. Regarding age-dependent hemodynamic differences, we applied a prewhitening process to address this issue. In addition to the fNIRS-related considerations, it’s important to acknowledge a limitation of this study, which is the smaller number of EL infants compared to TL infants. Given that one of the primary objectives of this study was to investigate mother–infant brain coupling during early development, we included a substantial number of typically developing infants. Furthermore, we did not exclude TL participants to balance the numbers between the two groups because statistical detection power improves with a larger number as well as for ANOVA. Nevertheless, a greater number of EL infants are desirable in future studies. Another limitation of this study is the potential difference in drowsiness depending on the condition, which we were unable to control. Specifically, it is possible that there was lower drowsiness (higher arousal) during breastfeeding and higher drowsiness during the holding condition, as infants were required to engage in at least some active movement during breastfeeding. This heightened arousal level may have contributed, in part, to the strong neural coupling observed in the breastfeeding condition. Conducting EEG measurements to assess arousal levels could be considered a potential solution for future studies.

Using fNIRS hyperscanning of mothers and 3–4-month-old infants, the present study revealed inter-brain coupling that is a neural underpinning of the mother–infant bonding in early development. Among the breastfeeding and holding conditions, breastfeeding, a fundamental mother–infant interplay, was exclusively associated with synchronous brain activities in which the cerebral foci differed between mothers and infants: the ventral part of the prefrontal cortex, particularly the R-OFC, for the mothers and the TPJ area, with a focus on L-STG/STS, for the infants. These inter-brain networks were considered to be involved with the reward-motivation system for the OFC and the mentalization system for the TPJ because these synchronizations were correlated with several measures related to attachment and social behaviors. Notably, these results did not differ between the two infant groups (TL and EL groups), although the EL group showed a stronger synchronization. For the inter-brain coupling underlying breastfeeding that does not require much social prediction, infants’ early mentalizing systems may have functioned similarly in both groups to coordinate the breastfeeding movement by predicting the mother’s movement. This study is the first to reveal that differential social attachment neural systems in the mother and infant brains connect to form inter-brain synchrony in early infancy using fNIRS with high spatial resolution. The neural system of attachment based on the R-OFC within the mother’s brain has been shown to contribute to neural coupling with the infant’s brain.

## Supplementary Material

Supplementary_bhad395

SupplementaryPrint_bhad395

## Data Availability

Due to the ethical restriction, the data cannot be made open access to the public. However, the data could be available from the corresponding author upon reasonable request.

## CRediT author statement

Yasuyo Minagawa (Conceptualization, Data curation, Formal analysis, Funding acquisition, Investigation, Methodology, Project administration, Resources, Supervision, Validation, Visualization, Writing—original draft, Writing—review & editing), Masahiro Hata (Data curation, Investigation), Eriko Yamamoto (Data curation, Investigation), Daisuke Tsuzuki (Data curation, Methodology, Software), Satoshi Morimoto (Data curation, Formal analysis, Methodology, Visualization, Writing—review & editing).
