# Supplementary material for: Inter-brain synchrony during mother–infant interactive parenting in 3–4-month-old infants with and without an elevated likelihood of autism spectrum disorder
Source: Cereb Cortex. 2023 Oct 26;33(24):11609–22. doi: 10.1093/cercor/bhad395 (PMC10724871; doi:10.1093/cercor/bhad395)
Supplement: Supplementary_bhad395 [file supplementary_bhad395.pdf]

Supplementary materials

Table S1. Significant main effects for the condition factor obtained from mother-infant synchronization. Significant results of the post-hoc test are also indicated. Abbreviations: BF, breastfeeding; H, holding; C, control; FDR corrected, Q

No.	Channel (Mother)	Channel (Infant)	d.f. Condition	d.f. Error	F-value	p-value	Q-value	partial eta squared	Post-hoc test: significant results
1	13	24	2	137	14.06	0.000003	0.0018	0.17	BF>H, BF>C
2	1	10	2	133	12.25	0.000013	0.004	0.156	BF>H, BF>C
3	2	10	2	152	12.2	0.000012	0.004	0.138	BF>H, BF>C
4	32	16	2	155	11.44	0.000023	0.0049	0.129	BF>H, BF>C
5	23	37	2	156	11.43	0.000023	0.0049	0.128	BF>H, BF>C
6	10	10	2	147	11.21	0.00003	0.0053	0.132	BF>H, BF>C
7	23	42	2	131	11.29	0.00003	0.0053	0.147	BF>H, BF>C
8	8	14	2	125	10.64	0.000054	0.0081	0.145	BF>H, BF>C
9	5	10	2	148	10.1	0.000077	0.009	0.12	BF>H, BF>C
10	10	28	2	143	9.95	0.00009	0.01	0.122	BF>H, BF>C
11	29	16	2	141	9.86	0.000099	0.0104	0.123	BF>H, BF>C
12	35	42	2	112	9.83	0.000117	0.0116	0.149	BF>H, BF>C
13	23	38	2	167	9.37	0.000139	0.0116	0.101	BF>H, BF>C
14	32	17	2	146	9.15	0.00018	0.0132	0.111	BF>H, BF>C
15	23	40	2	162	9.11	0.000178	0.0132	0.101	BF>H, BF>C
16	9	10	2	148	8.91	0.000222	0.0151	0.107	BF>H, BF>C
17	23	7	2	159	8.71	0.000257	0.016	0.099	BF>H, BF>C
18	23	10	2	158	8.73	0.000253	0.016	0.1	BF>H, BF>C
19	24	40	2	153	8.41	0.000343	0.019	0.099	BF>H, BF>C
20	27	43	2	162	8.29	0.000375	0.0197	0.093	BF>H, BF>C
21	3	20	2	147	8.18	0.000428	0.0216	0.1	BF>H, BF>C
22	29	7	2	135	8.17	0.000447	0.0216	0.108	BF>H, BF>C
23	5	20	2	142	8.12	0.000457	0.0216	0.103	BF>H, BF>C
24	3	8	2	148	8	0.000503	0.0229	0.098	BF>H, BF>C
25	1	37	2	135	8.04	0.000504	0.0229	0.106	BF>H
26	3	37	2	154	7.99	0.0005	0.0229	0.094	BF>H, BF>C
27	13	17	2	135	7.8	0.000621	0.0262	0.104	BF>H, BF>C
28	8	35	2	140	7.67	0.000688	0.0281	0.099	BF>H, BF>C
29	17	17	2	98	7.8	0.00072	0.0284	0.137	BF>H, BF>C
30	25	39	2	127	7.68	0.000713	0.0284	0.108	BF>H, BF>C

31	25	43	2	126	7.66	0.000727	0.0284	0.108	BF>H, BF>C
32	31	10	2	135	7.6	0.000744	0.0286	0.101	BF>H, BF>C
33	25	10	2	123	7.48	0.000859	0.0316	0.108	BF>H
34	10	15	2	152	7.36	0.000889	0.032	0.088	BF>H, BF>C
35	27	16	2	159	7.36	0.000881	0.032	0.085	BF>H, BF>C
36	3	7	2	155	7.3	0.000933	0.0324	0.086	BF>H
37	9	43	2	152	7.31	0.00093	0.0324	0.088	BF>H
38	10	14	2	134	7.23	0.001038	0.0346	0.097	BF>H, BF>C
39	9	15	2	154	7.2	0.001028	0.0346	0.085	BF>H, BF>C
40	13	10	2	143	7.14	0.001108	0.0361	0.091	BF>H, BF>C
41	28	16	2	143	7.1	0.001144	0.0369	0.09	BF>H
42	4	13	2	154	7.05	0.00118	0.0371	0.084	BF>H, BF>C
43	26	43	2	125	7.12	0.001177	0.0371	0.102	BF>H, BF>C
44	9	32	2	159	7.01	0.001207	0.0376	0.081	BF>H, BF>C
45	26	10	2	125	7.08	0.00122	0.0377	0.102	BF>H
46	8	13	2	133	7.03	0.001252	0.038	0.096	BF>H, BF>C
47	23	43	2	163	6.95	0.001275	0.0384	0.079	BF>H
48	14	10	2	97	7.11	0.001311	0.0388	0.128	BF>H, BF>C
49	23	24	2	151	6.91	0.001341	0.0388	0.084	BF>H, BF>C
50	27	38	2	166	6.89	0.00134	0.0388	0.077	BF>H
51	24	7	2	153	6.89	0.001364	0.0389	0.083	BF>H, BF>C
52	23	33	2	172	6.86	0.001359	0.0389	0.074	BF>H
53	27	40	2	161	6.77	0.001497	0.042	0.078	BF>H, BF>C
54	9	28	2	147	6.68	0.001678	0.0457	0.083	BF>H
55	3	10	2	155	6.51	0.001933	0.0493	0.077	BF>H, BF>C
56	32	39	2	155	6.52	0.001914	0.0493	0.078	BF>H
57	24	16	2	153	6.49	0.001963	0.0496	0.078	BF>H, BF>C

Table S2. Statistical results of correlation analysis between WTC and various behavioural measures (A-D). The WTC values used here represent the difference in WTC (subtracted value) between breastfeeding (BF) and one of the other two conditions (H = holding, C = control) as indicated in the "WTC condition". (A) Questionnaire-based measures include reward dependency in mothers (RDTm) and infants (RDTi), and postpartum bonding quotient (PBQ) at 3–4 months of age. (B) General development assessments from ESIAD include language score (LS) at 3–4 months of age. (C) Behavioural coding results during SFP, including vocalisation during the free-play phase (P-vocal), vocalisation during the still-face phase (S-vocal), and crying or fussing during the still-face phase (S-cry). (D) Score for expressive vocabulary at 18 months of age. Note that the number of data sets differed for each analysis because of differences in valid WTC data and each behavioural data depending on participants.

(A) Bonding and Temperament Questionnaires

Ch. Mother	Ch. Infant	WTC Condition	Index	N	r	p-value
5	20	BF-C	RDTi	22	0.53	0.0118
10	10	BF-C	RDTm	26	0.47	0.0157
5	20	BF-R	RDTi	26	0.46	0.0169
3	10	BF-C	PBQ	27	-0.45	0.0194
10	10	BF-R	PBQ	32	-0.42	0.0157
3	37	BF-C	RDTi	33	0.40	0.0198
24	40	BF-R	RDTi	41	0.39	0.0110

(B) Developmental Questionnaires

Ch.Mother	Ch.Infant	WTC Condition	Index	N	r	p-value
28	16	BF-C	LS	27	0.56	0.0026
2	10	BF-R	LS	37	0.51	0.0013

(C) Behavioural measures obtained from still face paradigm

Ch. Mother	Ch. Infant	WTC Condition	Index	N	r	p-value
23	42	BF-R	S_vocal	16	-0.66	0.0056
23	42	BF-R	S_cry	16	0.63	0.0093
8	13	BF-R	S_cry	22	0.56	0.0062
32	39	BF-C	P_vocal	31	0.51	0.0031
23	38	BF-C	P_vocal	36	0.43	0.0083

(D) Language development at 18 months of age

Ch. Mother	Ch. Infant	WTC Condition	Index	N	r	p-value
2	10	BF-C	Vocabulary	10	0.83	0.0032
9	28	BF-C	Vocabulary	14	0.73	0.0029
23	37	BF-R	Vocabulary	15	0.71	0.0028

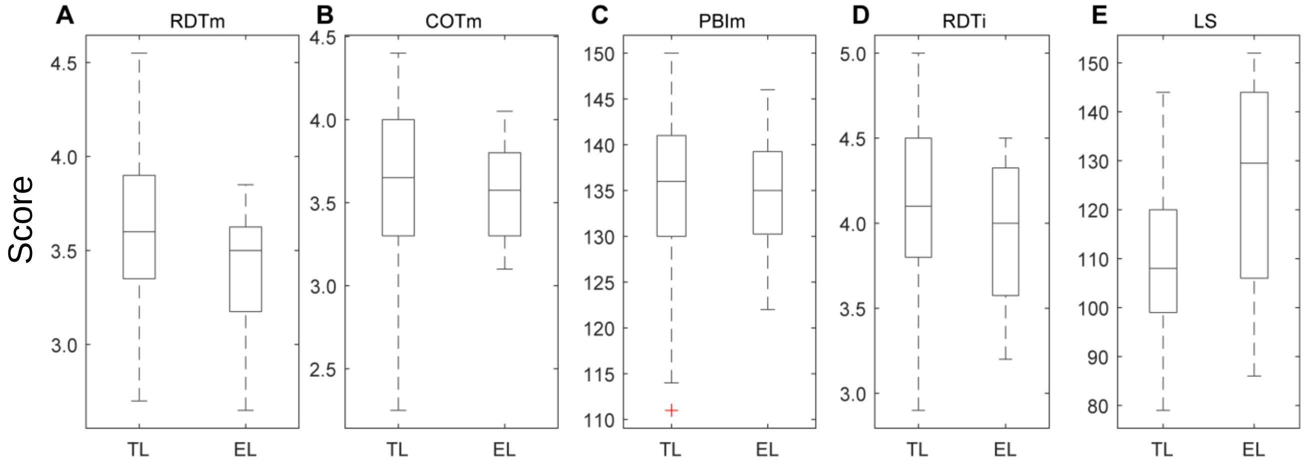

Supplementary Fig S1. Group differences in the Questionnaire-based score

Boxplot shows the scores of TL and EL groups for each questionnaire (A: reward dependency of mothers from TCI, RDTm; B: cooperativeness of mothers from TCI, COTm; C: postpartum bonding quotient, PBQ; D: reward dependency of infants from PSTCI, RDTi; E: language score from ESIAD, LS). No significant difference was observed.

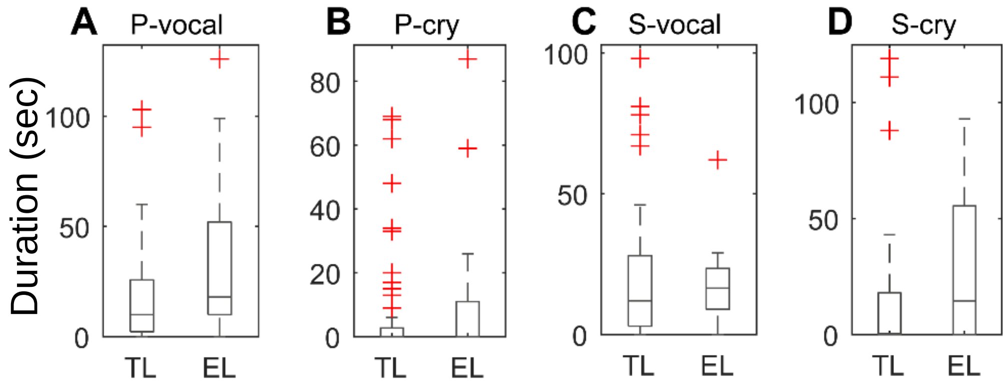

Supplementary Fig S2. Group differences in the SFP assessments

Boxplot shows the duration of the TL and EL groups for each infant's behaviour during the SFP assessment (A: positive vocalisation during the free-play (P) phase; B: crying during P phase; C: positive vocalization during the still-face (S) phase; D: crying during the S phase).

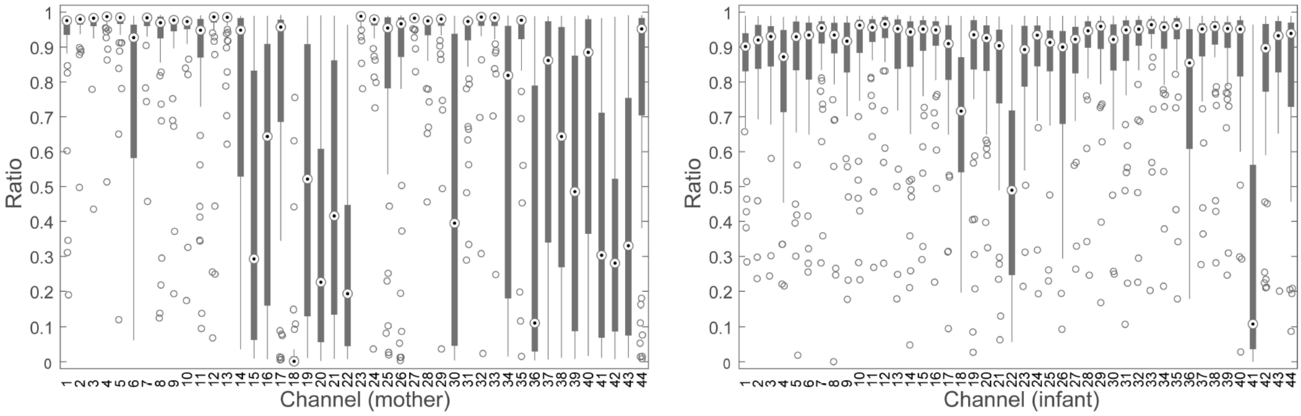

Supplementary Fig S3. Ratio of the valid sample periods of light intensity data

Boxplots show the ratio of available data duration as a result of the PHOEBE application for each channel in the mother (left) and infant (right). Note that channels 15,18, 20,21,22,30,36,39,41,42, and 43 for the mother's channel and 22 and 41 for the infant's channel were excluded from the analysis.

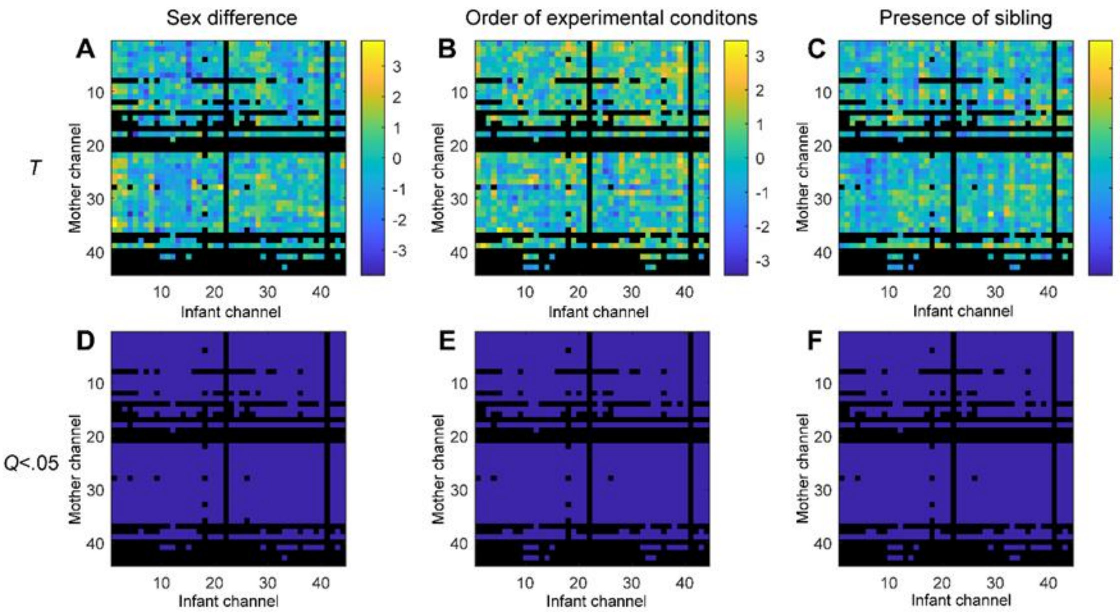

Supplementary Fig S4. Effect of external variables on the WTCs for all participants

Upper panels (A-C) show the T-values by colour, and lower panels (D-F) show the presence of significance by colour (blue: n.s. (Q<.05)). For each panel, the horizontal axis indicates the infant channels and the vertical axis indicates the mother channels. Black indicates data excluded due to noise factors. Note that there were no significant channel pairs.

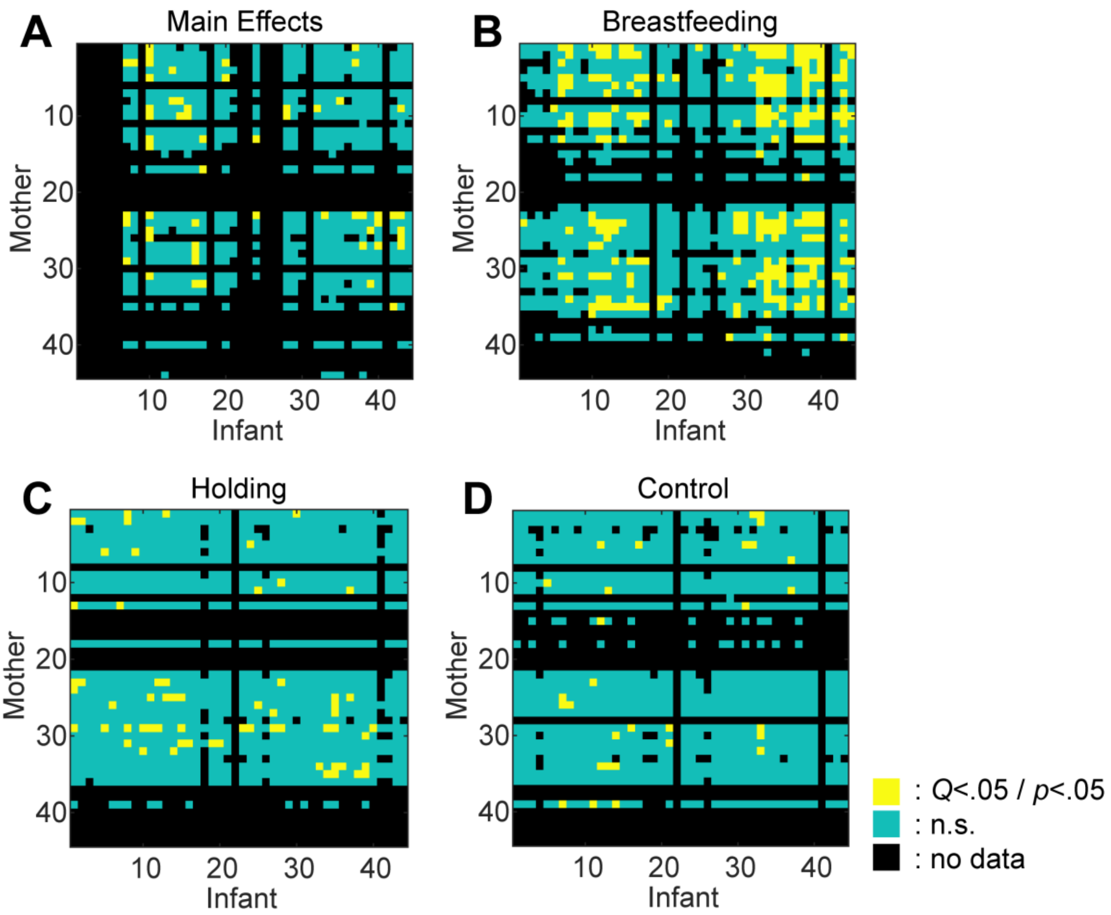

Supplementary Fig S5. Significant WTCs in ANOVA analysis (A) and comparison with baseline data in different conditions (B-D)

The main effects of the condition are presented by F-values (A). Results of the permutation test (t-values) are shown for the breastfeeding condition (B), the holding condition (C), and the control condition (D). For each panel, the horizontal axis indicates the infant channels and the vertical axis indicates the mother channels. Black indicates excluded data due to noise factors.
